# Heterogeneity of the Peripheral Circadian Systems in *Drosophila melanogaster*: A Review

**DOI:** 10.3389/fphys.2016.00008

**Published:** 2016-01-29

**Authors:** Chihiro Ito, Kenji Tomioka

**Affiliations:** Department of Biological Science, Division of Earth, Life, and Molecular Sciences, Graduate School of Natural Science and Technology, Okayama UniversityOkayama, Japan

**Keywords:** circadian rhythm, circadian clock, cryptochrome, *Drosophila*, peripheral oscillator, physiological rhythms, molecular oscillatory mechanism

## Abstract

Circadian rhythms in organisms are involved in many aspects of metabolism, physiology, and behavior. In many animals, these rhythms are produced by the circadian system consisting of a central clock located in the brain and peripheral clocks in various peripheral tissues. The oscillatory machinery and entrainment mechanism of peripheral clocks vary between different tissues and organs. The relationship between the central and peripheral clocks is also tissue-dependent. Here we review the heterogeneous nature of peripheral circadian clocks in the fruit fly *Drosophila melanogaster* and their dependence on the central clock, and discuss their significance in the temporal organization of physiology in peripheral tissues/organs.

Circadian rhythms are often observed in animal behavior, physiology, and gene expression (Aguilar-Roblero et al., 2015). In the fruit fly, *Drosophila melanogaster*; *one* of the most useful model organisms for the study of the circadian system, the central clock consists of about 150 clock gene expressing neurons in the brain (Taghert and Shafer, 2006; Shafer and Yao, 2014). The central clock regulates behavioral rhythms including locomotor behavior and sleep-wake cycles and is clustered in two neural groups, lateral neurons (LNs) and dorsal neurons (DNs). LNs are subdivided into three clusters, small ventral LNs (s-LNv), large ventral LNs (l-LNv), and dorsal LNs (LNd). DNs are subdivided into three clusters, DN_1_, DN_2_, and DN_3_(reviewed by Helfrich-Förster, 2005; Hermann-Luibl and Helfrich-Förster, 2015). The s-LN_v_ are necessary and sufficient for sustained locomotor rhythm in constant darkness (DD) (Helfrich-Förster, 1998; Renn et al., 1999) and are the most important cluster in the central circadian network (Grima et al., 2004; Stoleru et al., 2004, 2005). The dynamic neuronal network between the clock neurons is regulated by various neurotransmitters including pigment dispersing factor (PDF), which is expressed in the s- and l-LNvs (Shafer and Yao, 2014; Yao and Shafer, 2014; Hermann-Luibl and Helfrich-Förster, 2015) and fine-tunes the circadian rhythm to adapt to the environmental cycles (Miyasako et al., 2007; Yao and Shafer, 2014).

In addition to the central clock, peripheral clocks reside in various organs and tissues and likely regulate rhythms in organ/tissue specific functions (Table 1). Some peripheral clocks have been characterized only by immunohistochemistry to detect the oscillation of clock proteins or by reporter assays with luciferase expression driven by clock gene promotors (Giebultowicz and Hege, 1997; Plautz et al., 1997). Molecular studies revealed that the peripheral clocks are based on cell-autonomous molecular oscillation and that most directly respond to light when kept in culture conditions (Plautz et al., 1997). Further detailed studies focusing on molecular oscillations in the periphery and their output rhythms have revealed diversity in the circadian organization among the peripheral circadian systems (e.g., Myers et al., 2003; Ito et al., 2008; Krupp et al., 2013). In the present review, we summarize and discuss the features of peripheral oscillators, their heterogeneity, and their relationship to the central clock.

**Table 1:** **Peripheral clocks in *Drosophila melanogaster***.

**Location of clock**		**Output rhythm/relevant physiology**	Relationship to the central oscillator^*^	**References**
Chemosensory hairs	Antenna	−	A	Plautz et al., 1997
	Proboscis	−	A	Plautz et al., 1997
	Legs	−	A	Plautz et al., 1997
	Wing margin	−	A	Plautz et al., 1997
Excretory organs	Malpighian tubules	−	A	Giebultowicz and Hege, 1997; Giebultowicz et al., 2001
Digestive organs	Alimentary canal (esophagus, crop, proventriculus, hind gut, and rectum)	−	−^*^	Giebultowicz et al., 2001
Reproductive organs	Sparmathecae (female)	−	−^*^	Giebultowicz et al., 2001
	Paraovaria (female)	−	−^*^	Giebultowicz et al., 2001
	Testis base (male)	Sperm release	−^*^	Giebultowicz et al., 2001; Beaver et al., 2002
	Seminal vesicle (male)	Sperm release	−^*^	Giebultowicz et al., 2001; Beaver et al., 2002
	Ejaculatory ducts (male)	Sperm release	−^*^	Giebultowicz et al., 2001; Beaver et al., 2002
Visual system	Retina (compound eyes)	Electroretinogram	−^*^	Chen et al., 1992
Sensory neurons	Antenna (antennal neurons)	Olfaction rhythm	A	Krishnan et al., 1999; Tanoue et al., 2004
	Proboscis (gustatory receptor neurons)	Rhythms in gustatory physiology and behavior	A	Chatterjee et al., 2010
Epidermis	Epidermal cells	Cuticle deposition rhythm	A	Ito et al., 2008
Secretory cell	Oenocytes	Sex pheromone synthesis and emission	B	Krupp et al., 2008, 2013
Energy metabolic system	Fat body	Feeding rhythm	Possibly A	Xu et al., 2008, 2011
Endocrine system	Prothoracic gland	Eclosion rhythm	C	Emery et al., 1997; Myers et al., 2003; Morioka et al., 2012

* *See Figure 1B*.

*(-) Oscillations for clock protein or clock gene expression are observed. Final output rhythm is not yet determined*.

(−^*^) unknown.

## Heterogeneity in molecular machinery: The function of CRY in peripheral clocks

The circadian oscillation in central clock neurons is based on transcriptional/translational feedback loops that involve the transcription factors CLOCK (CLK) and CYCLE (CYC) (Tataroglu and Emery, 2015). CLK and CYC dimerize and induce transcription of *period* (*per*) and *timeless* (*tim*) genes. The translated products, PER and TIM, form a heterodimer that suppresses the activity of CLK-CYC to produce rhythmic expression of *per* and *tim* with a period of about 24 h. Light input causes degradation of TIM via the blue light receptor, CRYPTOCHROME (CRY), causing the circadian clock to be reset (Stanewsky et al., 1998; Suri et al., 1998; Ceriani et al., 1999; Busza et al., 2004). In addition to CRY, the visual system, including compound eyes, ocelli, and Hofbauer-Buchner eyelets, are involved in photic entrainment of the central clock (Stanewsky et al., 1998; Helfrich-Förster et al., 2001).

**Figure 1:**
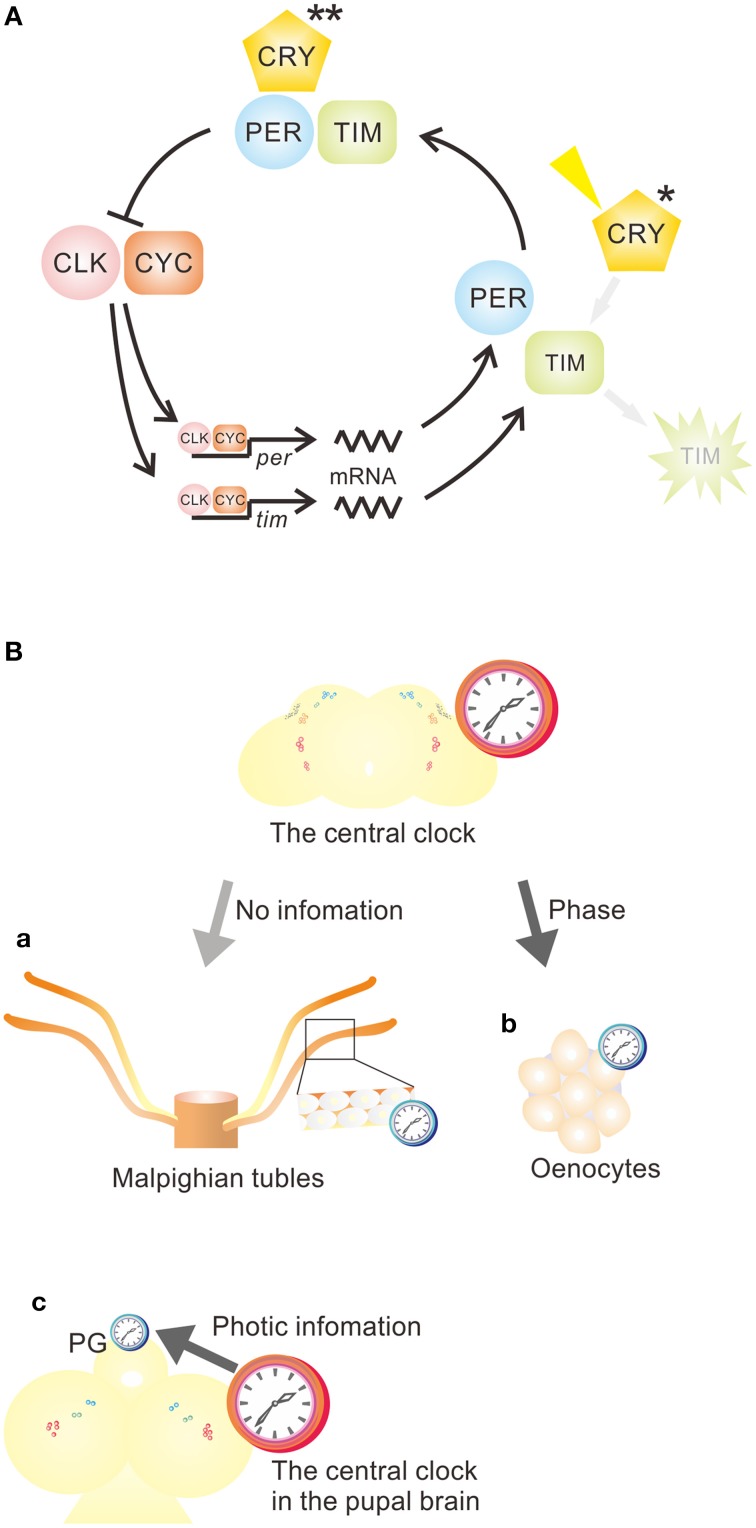
**Function of CRY in peripheral circadian clocks and the relationship between central and peripheral clocks in *Drosophila melanogaster***. **(A)** The functions of CRY vary in peripheral circadian clocks. In most peripheral circadian systems, CRY functions as a photoreceptor (^*^) and a core component (^**^) of the clock. However, CRY acts as a photoreceptor (^*^), but not as a core component of the clock, in the epidermis, which controls cuticle deposition rhythm, and in the prothoracic gland (PG). CLK, CLOCK; CRY, CRYPTOCHROME; CYC, CYCLE; *per, period*; *tim, timeless*. **(B)** Various relationships between central and peripheral clocks. **(a)** Most peripheral oscillators are independent of the central clock. **(b)** Some peripheral oscillators, such as oenocyte oscillators, are a slave to the central clock, receiving phase information to maintain an appropriate phase relationship to the central clock. **(c)** Some peripheral oscillators, such as those in PG, receive light and temporal signals from the central clock to drive oscillation and coordinate molecular oscillation. See Table 1 for more examples.

The oscillatory mechanism is shared by the central and the peripheral oscillators (Hardin et al., 2003); however, the function of CRY varies between peripheral oscillators: two different roles for CRY in the periphery have been suggested. (1) CRY functions as a photoreceptor and a core clock component (Stanewsky et al., 1998; Ivanchenko et al., 2001; Collins et al., 2006), and (2) CRY serves only as a photoreceptor (Ito et al., 2008) (Figure 1A). The following two sections provide examples for tissues/organs within which CRY has different roles.

### CRY serves as both a photoreceptor and a clock component

The dual roles of CRY as a photoreceptor and a clock component are well-demonstrated in Malpighian tubules (MT) (Stanewsky et al., 1998; Ivanchenko et al., 2001). In this organ, a light pulse given during the subjective night induces the degradation of TIM, resulting in a reset of the phase of circadian oscillation. Light-induced TIM degradation was eliminated in *cry*^*b*^, hypomorphic mutants lacking functional CRY. In addition, the PER and TIM oscillations in the MT disappeared in *cry*^*b*^ mutants (Stanewsky et al., 1998; Ivanchenko et al., 2001). These studies suggest that CRY has roles both in light entrainment and in the molecular oscillatory machinery of the clock. Another example is the antenna. Its response to odorants, as measured by an electroantennogram (EAG), increases at night and decreases during the day under light-dark (LD) cycles (Krishnan et al., 1999). The olfactory EAG rhythm is driven by a peripheral circadian clock in antennal olfactory sensory neurons, persisting under constant darkness (DD) (Krishnan et al., 1999; Tanoue et al., 2004). The rhythm is based on the molecular oscillatory mechanism and is abolished in *per* and *tim* null mutants (Krishnan et al., 1999). The circadian rhythms in olfactory EAG responses and clock gene expressions were both eliminated in *cry*^*b*^ mutant flies (Stanewsky et al., 1998; Krishnan et al., 2001). One might argue that the loss of rhythm is derived from desynchronization among constituent clock cells caused by a loss of photic entrainability. However, it is more likely that CRY serves as an essential component for the oscillatory machinery (Krishnan et al., 2001; Levine et al., 2002; Collins et al., 2006). CRY may function as a transcriptional repressor together with PER, because overexpression of *cry* and *per* repressed CLK-CYC activity in the compound eyes. The function of CRY as a transcriptional repressor was also confirmed in cultured cells (Collins et al., 2006). Thus, CRY's repressor activity explains why most peripheral clocks lose their oscillation in *cry*^*b*^ mutants (Levine et al., 2002).

### Cry serves only as a photoreceptor

In some peripheral rhythms, CRY seems to serve only as a photoreceptor for photic entrainment of the clock. One good example is cuticle deposition rhythms. In *Drosophila*, cuticle deposition rhythmically occurs in the endocuticle of the furca, an apodemata in the thorax, and the rhythm is controlled by a peripheral circadian clock residing in epidermal cells (Ito et al., 2008). The cuticle deposition rhythm in furca was entrained to LD cycles even when the thorax was cultured *in vitro*, suggesting that the photic entrainment system is independent of the brain and resides in the thorax. In *cry*^*b*^ mutants, the rhythm was not entrained to LD cycles. The entrainability was rescued by the overexpression of *cry* throughout whole body of *cry*^*b*^ mutants. Interestingly, not only *cry*^*b*^, but also *cry*^*OUT*^ knockout mutants completely lacking CRY, exhibit the free-running rhythm of cuticle deposition (Ito et al., 2008). These results suggest that CRY only functions as a photoreceptor and not as a component of the clock machinery in the cuticle deposition rhythm.

A similar function of CRY is seen in the prothoracic gland (PG). Fruit fly adults emerge from the pupal case around dawn (Konopka and Benzer, 1971), when high humidity is thought to protect newly emerged flies from desiccation (Pittendrigh, 1954). This eclosion behavior is a once-in-a-lifetime event, but it occurs rhythmically in a population of flies at different developmental stages. The timing of eclosion is set by the circadian system, which consists of two oscillators: one in the LNv and another in the PG (Myers et al., 2003). Myers et al. (2003) suggested that CRY plays an important role as a core component of oscillatory machinery in the PG because the eclosion rhythm was abolished in *cry*^*b*^ mutants. However, subsequent studies yielded completely opposite results (Mealey-Ferrara et al., 2003; Dolezelova et al., 2007): the rhythmic eclosion was observed even in *cry*^*b*^ and *cry*^*0*^ (knockout of *cry*) flies both in LD cycles and DD. Therefore, as for the cuticle deposition rhythm in furca, CRY seems not to be involved in the oscillatory mechanism of the clock in the PG. This has been confirmed by molecular studies (Emery et al., 1997; Morioka et al., 2012). The *per* transcript rhythm persisted in the PG under DD when the central nervous system (CNS)-ring gland (RG) complex containing the PG was cultured. The *per* transcript rhythm and oscillation of TIM were both intact in cultured CNS-RG from *cry*^*b*^ mutant flies. However, the TIM oscillation seemed to free-run in PG cells isolated from a *cry*^*b*^ mutant kept under LD cycles (Morioka et al., 2012). Therefore, in the PG, CRY may play a role in the entrainment of TIM oscillation to LD cycles. Interestingly, *cry* expressed in the PG does not affect *per* oscillation. The *per* transcript levels in the PG increased when the cultured CNS-RG was exposed to 12 h of light. This response of *per* depended on light input from CRY via the CNS, because the response was eliminated by *cry*^*b*^ mutation, isolation of PG from the CNS, and blocking synaptic inputs from the CNS. The expression of *cry* transcripts was quite low in the PG compared with the brain and MT (Morioka et al., 2012). Taken together, it is likely that only a small amount of CRY is expressed in the PG and plays a role in the photic entrainment of TIM oscillation, but is not involved in the oscillatory machinery or the photic entrainment of *per* oscillation in the PG.

## Heterogeneity in circadian organization in the periphery

Although most peripheral oscillators can maintain their oscillation under *in vitro* culture conditions, the oscillations may not perfectly represent those *in vivo* where they may be influenced by various factors including those from the central clock. This issue has been addressed by several studies to date (Krishnan et al., 1999; Giebultowicz, 2000; Ivanchenko et al., 2001; Ito et al., 2008; Krupp et al., 2008, 2013; Morioka et al., 2012), and various relationships between the central and peripheral oscillators have been reported (Figure 1B).

### Peripheral clocks are independent of the central clock

The Malpighian tubules contain a peripheral clock (Giebultowicz and Hege, 1997) (Figure 1Ba). The peripheral oscillator in MT is cell-autonomous and entrained to environmental light without any cue from the central clock (Hege et al., 1997). Independency of the MT oscillator from the central clock was clearly revealed by Giebultowicz et al. (2000). They showed that the original phase of TIM oscillation of MT is maintained in DD when the MT is transplanted into the abdomen of flies previously entrained to antiphase LD cycles.

The antennal circadian clock is also independent from the central clock. The olfactory rhythm driven by the antennal clock was intact even when LNvs, the central clock neurons, were ablated or disrupted (Krishnan et al., 1999; Tanoue et al., 2004). However, the rhythm was lost in flies where the clock oscillation was disrupted only in olfactory sensory neurons (Tanoue et al., 2004). Thus, the peripheral clock in antennae is completely independent from the central clock.

The clock in the fat body of *Drosophila* is another example. It can be entrained independently of the central clock via a restricted feeding schedule (Xu et al., 2011). Interestingly, it has effects on fly metabolism that oppose the effect of the central clock: flies with a genetically disrupted fat body clock show increased food consumption, reduced levels of energy storage, and a higher sensitivity to starvation, whereas opposite responses are observed in energy storage and starvation when the central clock is disrupted (Xu et al., 2008).

### Peripheral oscillator is a slave to the central clock

Krupp et al. (2008) demonstrated that the circadian clock phase in oenocytes, which regulate pheromone production, is regulated by the central clock. The core clock genes, *per, tim, Clk*, and *cyc*, showed cyclic expression in LD cycles and DD in oenocytes. These cyclic expressions are abolished in *per*^*0*^ mutants in DD and in *per*7.2:2 transgenic flies, which only have PER expression in a subset of clock neurons in the brain, but not in peripheral tissues. The results suggest that oenocytes contain a *per*-dependent peripheral clock. The phase of clock gene expression is affected by PDF signaling: it is altered when PDF signaling is disrupted in mutants lacking PDF or a PDF receptor. In those mutants, however, clock gene expression is robustly rhythmic as in wild-type flies and the phase relationship among clock genes is maintained as normal (Krupp et al., 2013). This suggests that the peripheral oscillator in oenocytes is a slave oscillator and its phase is modulated by the central clock, although oscillation itself is maintained independent of the central oscillator (Figure 1Bb).

### Peripheral oscillator is driven by the central clock

The eclosion timing is controlled by a circadian system that consists of two hierarchically organized oscillators located in LNvs and PG, respectively (Myers et al., 2003; Morioka et al., 2012) (Figure 1Bc). The targeted disruption of either of these two circadian oscillators using *tim* overexpression renders the eclosion arrhythmic. The eclosion rhythm and molecular oscillation of TIM in PG are also diminished when LNvs are ablated (Myers et al., 2003). These results suggested that both LNv and PG clocks are necessary for eclosion rhythm and that the PG clock is a slave oscillator driven by the LNv clock. To further dissect this relationship, Morioka et al. (2012) observed the clock gene transcript rhythm and post-transcriptional rhythm in PG *in vitro* and found that the PER oscillation of PG clock receives light information from the central clock, but TIM oscillation does not. Interestingly, TIM maintains its oscillation, but PER does not in PG under DD, although both molecular oscillations are robust under LD cycles (Myers et al., 2003; Morioka et al., 2012). The control from the CNS may contribute to maintaining the robust coordinated oscillations of PER and TIM, which otherwise are dissociated from each other. Thus, the oscillator in *Drosophila* PG is governed by the central clock to a large extent.

## Conclusion and future directions

In early studies, the peripheral oscillators were suggested to be cell autonomous, directly light entrainable, and independent of the central oscillator (Plautz et al., 1997; Giebultowicz, 2000). As described above, new lines of evidence have clearly demonstrated that the oscillatory machinery and degree of independence from the central clock vary among the peripheral clocks. Why and how the peripheral circadian systems have diversified within a species is an open question. Unfortunately, no good answer to this question is currently available. In particular, the function of CRY as a repressor is the most challenging issue in this field. Mammals contain two CRYs, mCRY1 and mCRY2, both of which function as core clock components (Okamura et al., 1999; van der Horst et al., 1999). Many insects also contain two CRYs including *Drosophila* type dCRY and mammalian type mCRY. The former is suggested to be a blue light photoreceptor and the latter to be a transcriptional repressor (Zhu et al., 2005, 2008). *Drosophila* have only one CRY (dCRY) in the genome, which functions as a photoreceptor in the central circadian system (Stanewsky et al., 1998; Helfrich-Förster et al., 2001), but as a transcriptional repressor in the core clock machinery of some peripheral circadian systems (Collins et al., 2006). How the same molecule acts differently in the central and peripheral clocks should be elucidated in future studies. Interestingly, hymenopteran species, including honeybees, only contain mCRY in their genome (Rubin et al., 2006; Zhan et al., 2011). Because hymenopteran species lack *tim* gene in their genome, mCRY is suggested to function as a partner of PER (Rubin et al., 2006). The lack of dCRY might be related to a loss of TIM, because dCRY mediates light-dependent TIM degradation that is required for clock entrainment. It is thus likely that an ancestral insect had both dCRY and mCRY, and that during the course of evolution, some species retained both, while others lost either dCRY or mCRY (Yuan et al., 2007).

The difference between central and peripheral clocks can be also characterized by cell-to-cell communication. The central clock consists of a network of neurons communicating through peptidergic neurotransmitters and maintains its oscillation in various environmental conditions (Yao and Shafer, 2014), whereas communication in the peripheral clock may be variable in organs/tissues and the synchrony among cells is rapidly lost under constant conditions (Plautz et al., 1997; Morioka et al., 2012). Thus, in the latter, synchrony is largely maintained by environmental factors (Plautz et al., 1997; Giebultowicz et al., 2000) or by neuronal or humoral signals from the central clock (Morioka et al., 2012). Detailed molecular studies are necessary to understand the variability and importance of cellular communication in peripheral clock tissue.

Another important issue is the relationship between central and the peripheral clocks, which may be required to temporally optimize local physiology in a tissue-dependent manner. As discussed above, peripheral oscillators can be classified into three types in *Drosophila*: independent of, slave to, and driven by the central clock. Independent peripheral oscillators maintain their own phase, whereas the latter two need to be modulated by the central clock to temporally coordinate physiological and behavioral activity. Therefore, if control from the central clock is through humoral factors, such as ecdysteroids and PDF (Krupp et al., 2013; Uryu et al., 2015), it may be not be uniform, which might be attributable to different oscillatory mechanisms of peripheral clocks. Future studies are needed to examine the possible feedback from the peripheral oscillator to the central clock, which has already been shown in mammalian clock systems (Mohawk et al., 2012).

Although most of the studies on phase control of peripheral circadian clocks have focused on the role of light and the central oscillator, we should pay attention to environmental cues other than light. Obviously the peripheral oscillators utilize temperature changes as time cue (Zeitgeber) in addition to light. Interestingly, the temperature entrainment ability of the peripheral clock is eliminated in flies lacking *no receptor potential A* (*norpA*) encoding phospholipase C (Ito et al., 2011). Although *norpA* mediates thermosensitive splicing of *per* in the central clock (Majercak et al., 2004), its role in peripheral clocks remains to be elucidated. Another important Zeitgeber may be feeding (Xu et al., 2011): the detailed mechanism by which feeding entrains the peripheral oscillator should also be elucidated. Because the precise coordination of amplitude and phase of all clocks is essential for the well-being of animals (Hastings et al., 2003), it remains challenging to explore how multiple circadian clocks in the body are coordinated through entrainment by Zeitgebers and central-peripheral interactions.

## Author contributions

CI and KT developed the concept for this mini-review and CI prepared the early draft, including the figure and table. CI researched the literature for the key papers used in this mini-review. KT developed the early draft prepared by CI.

### Conflict of interest statement

The authors declare that the research was conducted in the absence of any commercial or financial relationships that could be construed as a potential conflict of interest.

## Abbreviations

CLK: CLOCK
*Clk*: *Clock*
CNS: central nervous system
CRY: CRYPTOCHROME
*cwo*: *clockwork orange*
CYC: CYCLE
*cyc*: *cycle*
DD: constant darkness
DN: dorsal neurons
EAG: electroantennogram
LD: light dark
LN: lateral neurons
LNv: ventral lateral neurons
s-LNv: small lateral neurons
l-LNv: large lateral neurons
LNd: dorsal lateral neurons
LPN: lateral posterior neurons
MT: Malpighian tubules
*norpA*: *no receptor potential A*
PDF: pigment dispersing factor
PER: PERIOD
*per*: *period*
PG: prothoracic gland
RG: ring gland
TIM: TIMELESS
*tim*: *timeless*.
